# Segmental Absence of Intestinal Musculature in Adults: A Report of Five Cases Among 86 Intestinal Perforations

**DOI:** 10.1155/crip/2027784

**Published:** 2026-07-23

**Authors:** Naoko Nambu, Yuki Kubo, Tsukasa Yoshikawa, Kaito Muroki, Nobuyuki Terada, Shin-ichi Nakatsuka

**Affiliations:** ^1^ Department of Pathology, Yao Tokushukai General Hospital, Yao, Osaka, Japan

**Keywords:** adult, case report, colon, perforation, segmental absence of intestinal musculature

## Abstract

Among 86 adult patients who underwent surgery for intestinal perforation, excluding cases caused by cancer invasion or appendicitis, diverticulitis (33/86, 38%) was identified as the most common cause of perforation, followed by ischaemia (7/86, 8%) and segmental absence of intestinal musculature (SAIM) (5/86, 6%). In patients with SAIM‐related perforation, the site of perforation was the sigmoid colon in four cases and the caecum in one. SAIM‐related perforation occurred predominantly in older women and most frequently involved the sigmoid colon. The four patients with sigmoid colon perforation showed an abrupt loss of the muscularis propria. In the patient with caecal perforation, the muscularis propria exhibited separation and thinning with focal complete loss. Secondary inflammation or haemorrhage associated with the perforation was observed in the bowel wall surrounding the perforation site. However, there was no evidence of primary suppurative inflammation or necrosis causing of perforation. Given that the prevalence of SAIM among patients with bowel perforation appeared to be higher than previously assumed, it is essential to histologically examine sufficient tissue samples to avoid missing this diagnosis. Recognition of SAIM is clinically important, especially in adults without previous or current evidence of intestinal disease, as it should be considered a possible cause of intestinal perforation, thereby facilitating early diagnosis and prompt treatment.

## 1. Introduction

Segmental absence of intestinal musculature (SAIM) is characterised by complete loss of the muscularis propria, with or without thinning, in a segment of the gastrointestinal tract [1–11]. SAIM was first recognised as a paediatric condition causing intestinal obstruction, perforation or intussusception, although this condition is rare [1–6]. Subsequently, cases in adults were reported, most commonly presenting with intestinal perforation; however, the number of reports remains limited [7–10], and SAIM is therefore generally considered a rare entity. In contrast, Tsuyuki et al. [11] reported that, among 109 cases of intestinal perforation, excluding those caused by cancer invasion or appendicitis, 26 (24%) were attributable to SAIM, suggesting a greater prevalence than previously assumed. In this study, we report five cases of SAIM that were identified through histological examination of surgical specimens from 86 patients with intestinal perforation.

## 2. Case Report

This study included surgical specimens from 86 patients who underwent surgery for intestinal perforation at Yao Tokushukai General Hospital between January 2023 and July 2025, excluding cases caused by cancer invasion or appendicitis. We reviewed all cases by examining macroscopic photographs stored in the electronic medical records and preserved haematoxylin and eosin (H&E)‐stained specimens to determine the cause of perforation. Cases without clear evidence of a specific cause were classified as having an unknown aetiology. SAIM was diagnosed when histological findings satisfied all three of the following criteria: (1) anatomically intact mucosa and muscularis mucosae in the bowel wall surrounding the perforation site; (2) absence of the muscularis propria, occasionally accompanied by variable degrees of thickening or structural abnormalities; and (3) absence of primary suppurative inflammation or necrosis causing the perforation (Table 1). Of these, five cases of intestinal perforation were attributed to SAIM. Among the 86 patients, 43 were female (range: 43–96 years; median: 82 years) and 43 were male (range: 25–88 years; median: 63 years). The cause of perforation was identified in 47 of the 86 patients, with diverticulitis identified as the most common cause (33/86, 38%), followed by ischaemia (7/86, 8%) and SAIM (5/86, 6%) (Table 2). The cause of intestinal perforation could not be determined in approximately half of the cases.

**Table 1:** Diagnostic criteria for SAIM.

1. Anatomically intact mucosa and muscularis mucosa at the wall surrounding the perforation site
2. Absence of muscularis propria, occasionally accompanied by structural abnormalities
3. Absence of primary suppurative inflammation or necrosis

Abbreviation: SAIM, segmental absence of intestinal musculature.

**Table 2:** Causes of intestinal perforation.

Cause	Number (%)
Diverticulitis	33 (38%)
Ischemia	7 (8%)
SAIM	5 (6%)
Amyloidosis	1 (1%)
Cholesterol embolism	1 (1%)
Unknown	39 (45%)

Abbreviation: SAIM, segmental absence of intestinal musculature.

Details of the five SAIM cases are presented in Table 3. Among the five patients, one was male and four were female, with ages ranging from 61 to 89 years. The site of perforation was the sigmoid colon in four patients and the caecum in one patient. The maximum diameter of the perforation ranged from 1.9 to 4.7 cm. Symptoms preceding perforation included sudden abdominal pain in three patients and pain extending from the chest to the back in one patient. One patient (Case 5), who had bowel obstruction due to sigmoid colon cancer, complained of abdominal bloating. In all cases, perforation was confirmed by abdominal computed tomography, either by the presence of free air in the abdomen or by the leakage of barium used during an upper gastrointestinal contrast examination. Overall, SAIM‐related perforations were observed predominantly in older women and most frequently involved the sigmoid colon.

**Table 3:** Clinical and pathological characteristics of the five patients with SAIM.

Patient no.	Sex	Age	Perforation site	Perforation diameter (max)	Symptom and CT findings	Muscularis propria and its nerve plexus	Areas surrounding the perforation site
1	F	80	Sigmoid	4.7 cm	Chest‐back pain, abdominal free air, ascites	Abrupt interruption, plexus (+)	Haemorrhage, food residue, neutrophil infiltration
2	F	61	Sigmoid	3.6 cm	Sudden abdominal pain, extraluminal contrast^a^	Abrupt interruption, plexus (+)	No haemorrhage, no inflammation
3	F	89	Sigmoid	1.9 cm	Sudden abdominal pain, abdominal free air	Abrupt interruption, plexus (+)	Submucosal haemorrhage, thin necrotic tissue layer with inflammatory cells
4	F	87	Sigmoid	2.8 cm	Sudden lower abdominal pain, abdominal free air, ascites	Abrupt interruption, plexus (+)	Intramuscular haemorrhage, thin layer of neutrophils at the submucosal base
5	M	70	Caecum	2.5 cm	Bloating in the abdomen for 2 weeks^b^, abdominal free air	Thinning or separation with eventual loss, plexus (‐)	Mild infiltration of lymphocytes, capillary proliferation

Abbreviations: CT, computed tomography; F, female; M, male; SAIM, segmental absence of intestinal musculature; Sigmoid, sigmoid colon.

^a^Patient (Case 2) underwent a barium test to check the upper digestive tract the day before.

^b^Patient (Case 5) had been diagnosed with bowel obstruction due to sigmoid colon cancer by CT 2 days before perforation.

**Figure 1:**
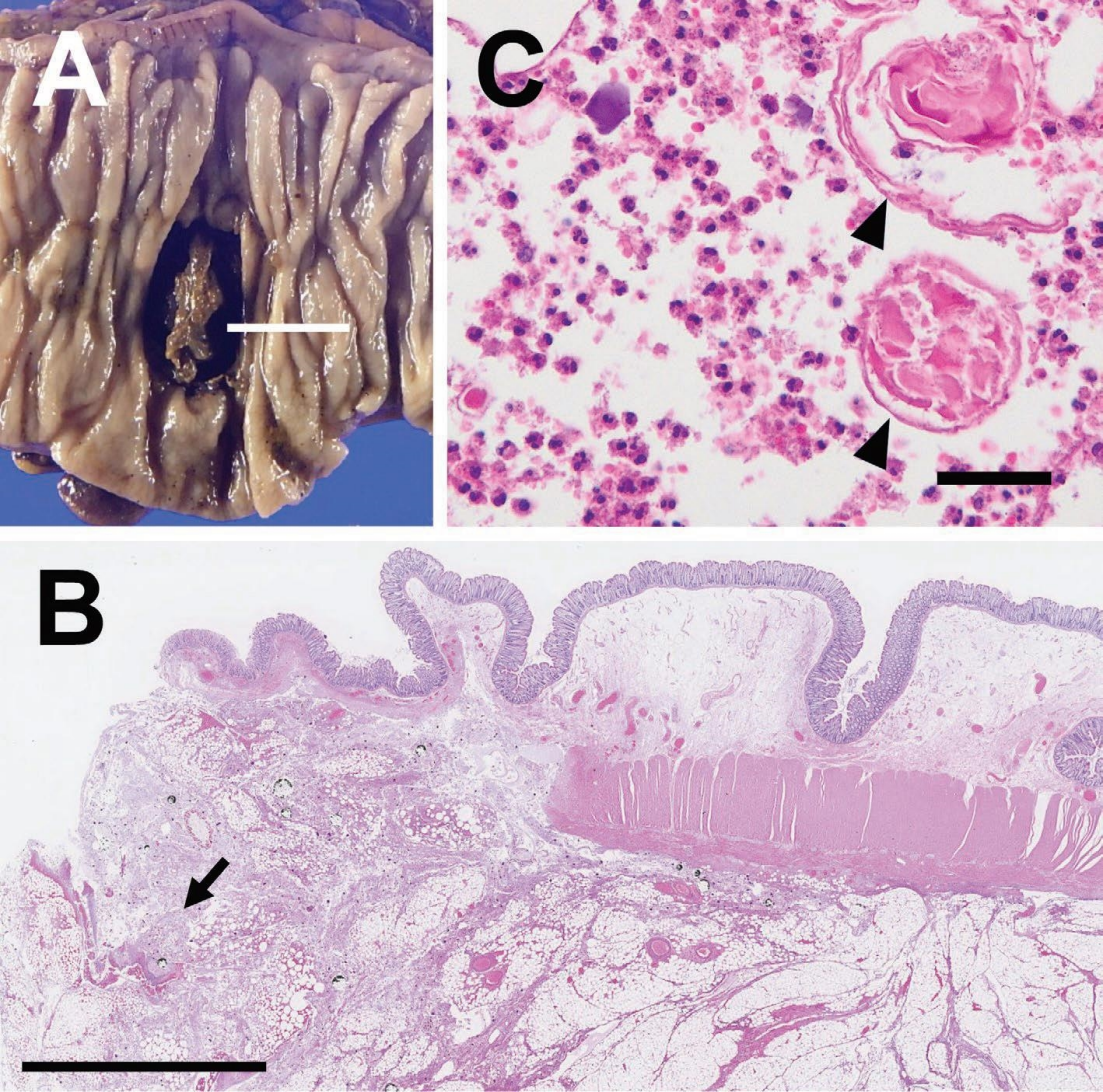
(A) Macroscopic view of the perforation in the sigmoid colon in Case 1. The white bar indicates the site from which the histological specimen was obtained. (B) Histological image of the specimen stained with haematoxylin and eosin (H&E). The muscularis propria shows abrupt loss. Scale bar: 5 mm. (C) Higher‐magnification view of the area indicated by the arrow in panel B. Food residues (arrowheads) and infiltration by segmented neutrophils are observed. Scale bar: 50 *μ*m.

**Figure 2:**
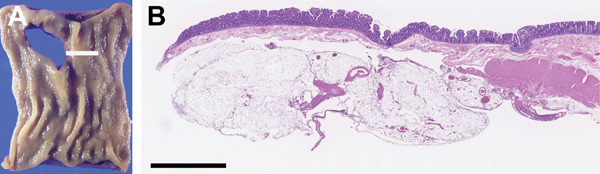
(A) Macroscopic view of the perforation in the sigmoid colon in Case 2. The white bar indicates the site from which the histological specimen was obtained. (B) Histological image of the specimen stained with H&E. The muscularis propria shows an abrupt thinning over a very short segment, followed by complete loss. Scale bar: 2.5 mm.

**Figure 3:**
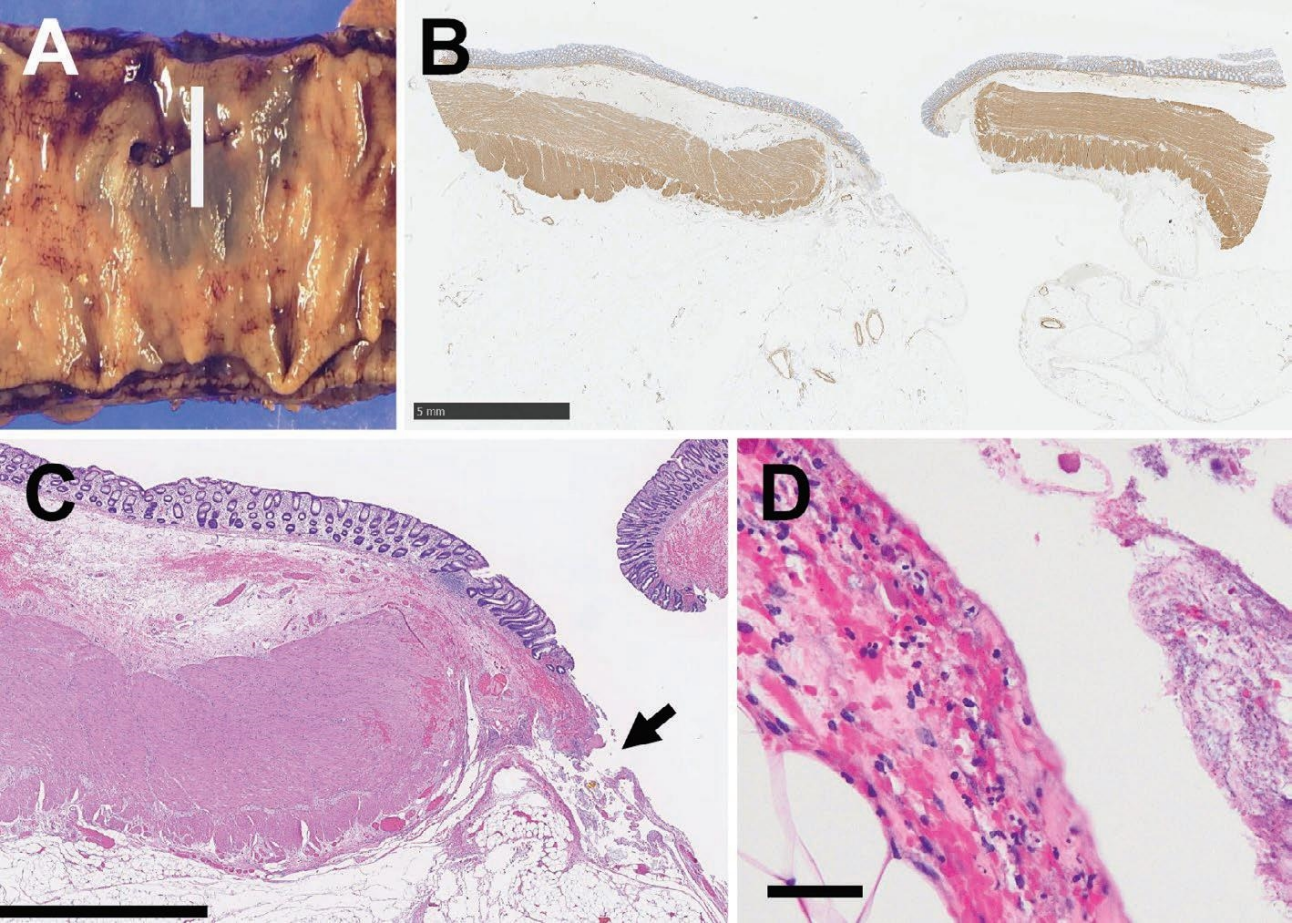
(A) Macroscopic view of the perforation in the sigmoid colon in Case 3. The white bar indicates the site from which the histological specimen was obtained. (B) Representative image showing immunohistochemical staining for *α*‐smooth muscle actin. The muscularis propria shows abrupt loss. Scale bar: 5 mm. (C) Histological image of the specimen stained with H&E. Scale bar: 2.5 mm. (D) Higher‐magnification view of the area indicated by the arrow in panel C. A thin layer of necrotic tissue is adherent to the wall of the perforation site. Scale bar: 50 *μ*m.

**Figure 4:**
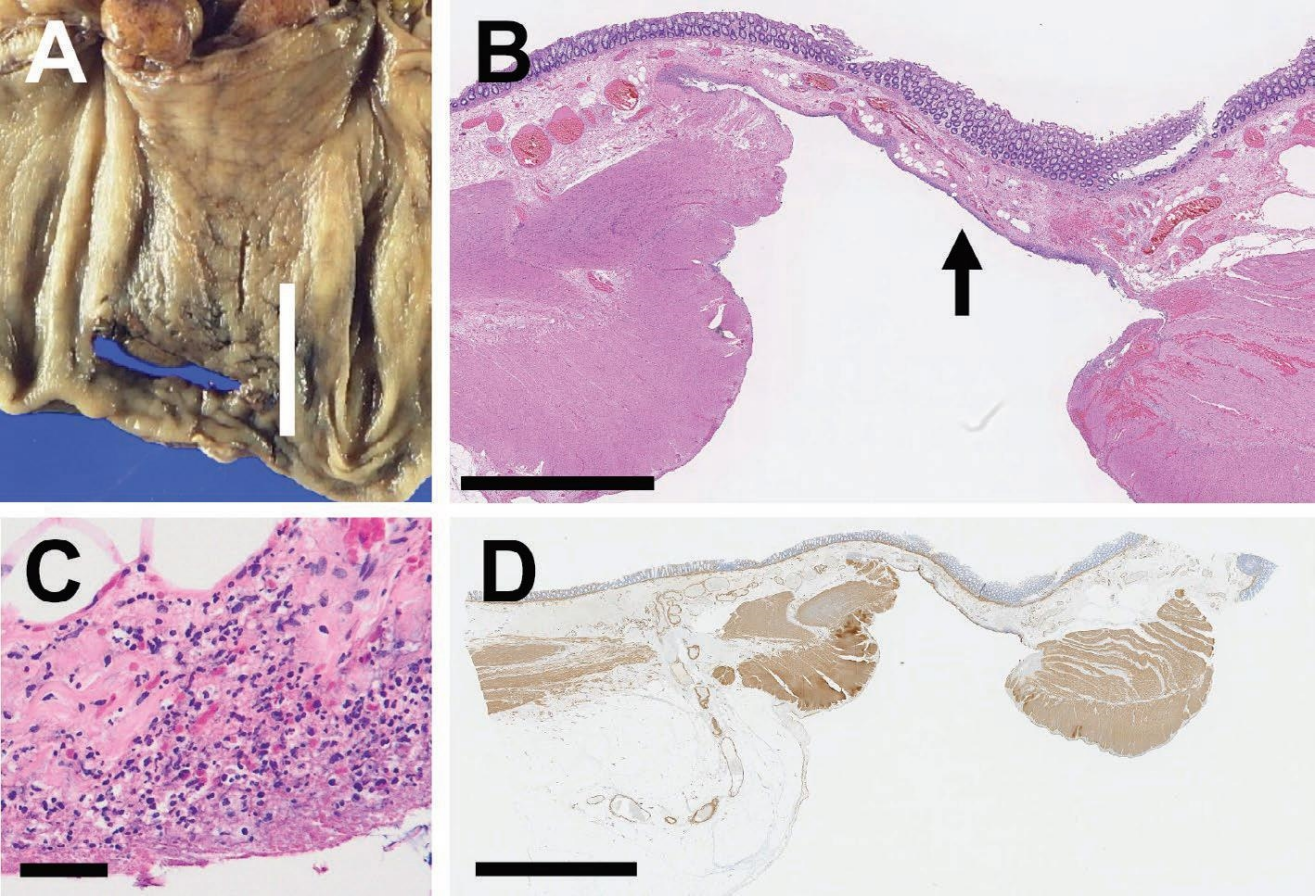
(A) Macroscopic view of the perforation in the sigmoid colon in Case 4. The white bar indicates the site from which the histological specimen was obtained. (B) Histological image of the specimen stained with H&E. Scale bar: 2.5 mm. (C) Higher‐magnification view of the area indicated by the arrow in panel (B). A thin layer of inflammatory cells, including segmented neutrophils and mononuclear cells, is observed. Scale bar: 50 *μ*m. (D) Representative image showing immunohistochemical staining for *α*‐smooth muscle actin. The muscularis propria exhibits focal complete loss with abrupt termination at its margins. Scale bar: 5 mm.

**Figure 5:**
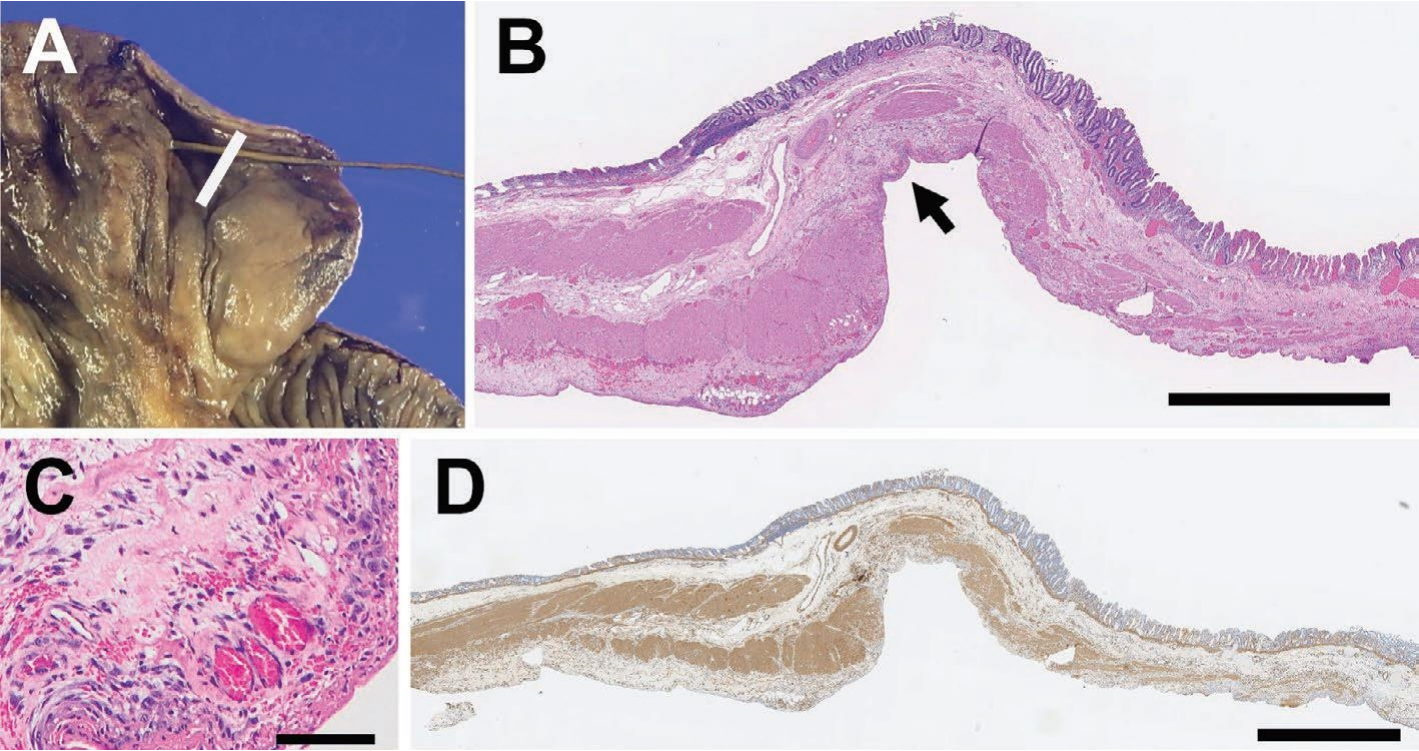
(A) Macroscopic view of the perforation in the caecum in Case 5. The white bar indicates the site from which the histological specimen was obtained. (B) Histological image of the specimen. The muscularis propria shows separation or thinning with focal complete loss on H&E staining. Scale bar: 2.5 mm. (C) Higher‐magnification view of the area indicated by the arrow in panel B. Representative image showing capillary proliferation accompanied by mild infiltration of mononuclear inflammatory cells. Scale bar: 50 *μ*m. (D) Representative image showing immunohistochemical staining for *α*‐smooth muscle actin. Scale bar: 2.5 mm.

Figures 1, 2, 3, 4 and 5 show the histological findings in the bowel wall surrounding the perforation site. In Cases 1 and 3, the muscularis propria showed an abrupt loss (Figures 1 and 3). In Case 2, the muscularis propria showed abrupt thinning over a very short segment, followed by complete loss (Figure 2). In Case 4, the muscularis propria exhibited focal complete loss with abrupt termination at its margins (Figure 4). In Case 5, the muscularis propria demonstrated separation or thinning, accompanied by focal complete loss (Figure 5).

**Figure 6:**
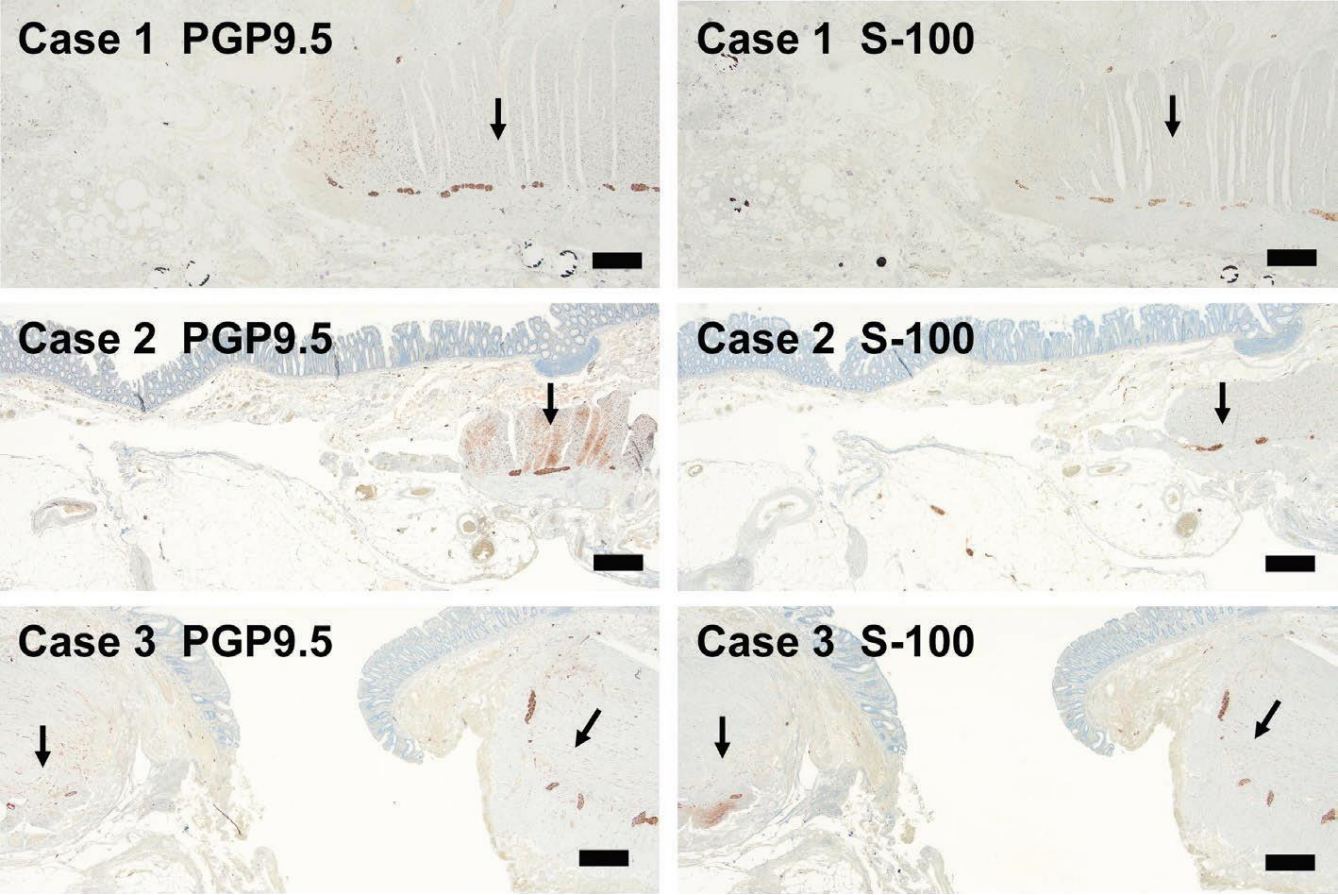
Immunohistochemical staining of tissue specimens from Cases 1, 2, and 3 for PGP9.5 (neuronal marker) and S‐100 (glial marker). Scale bar: 0.5 mm. Brown foci in the regions indicated by arrows represent positive immunostaining. In Cases 1, 2, and 3, the intermuscular nerve plexus containing both neural and glial cells is observed within the muscularis propria, but not in areas lacking the muscularis propria.

**Figure 7:**
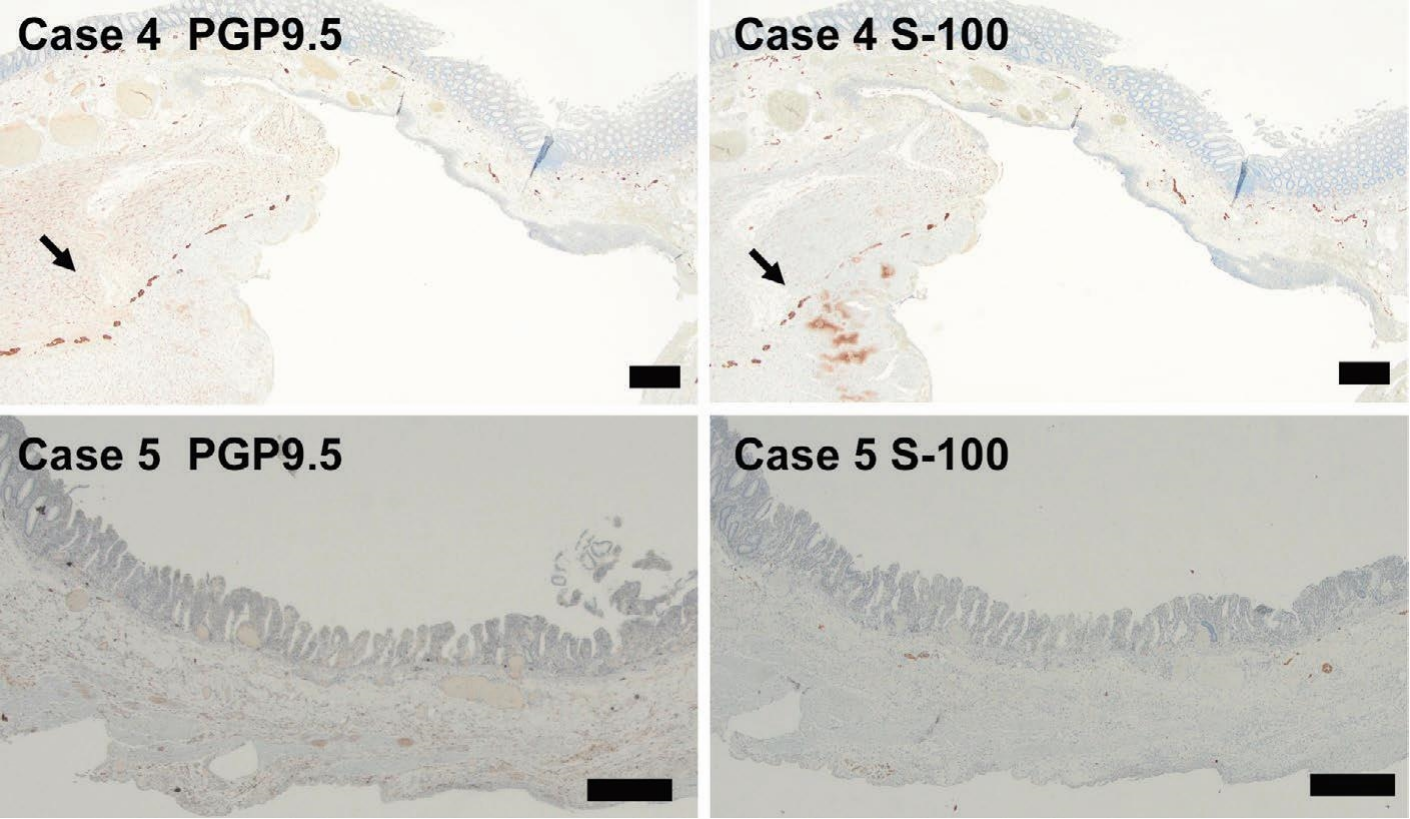
Immunohistochemical staining of tissue specimens from Cases 4 and 5 for PGP9.5 (neuronal marker) and S‐100 (glial marker). Scale bar: 0.5 mm. Brown foci in the regions indicated by arrows represent positive immunostaining. In Case 4, the intermuscular nerve plexus containing both neural and glial cells is observed within the muscularis propria; however, in Case 5, no such plexus is observed in areas with a thinned or absent muscularis propria.

Immunohistochemical staining with PGP9.5 (a neuronal marker) and S‐100 (a glial cell marker) revealed the presence of nerve plexuses within the muscularis propria in Cases 1–4, but not in areas where the muscularis propria was absent (Figures 6 and 7). In contrast, Case 5 showed no immunoreactivity for PGP9.5, whereas S‐100 immunoreactivity was detected either within the residual thinned muscularis propria or in areas showing complete loss of the muscularis propria (Figure 7).

In the area surrounding the perforation site in Case 1, numerous food residues were present, and haemorrhage, neutrophil infiltration and necrotic tissue fragments were observed in the adipose tissue of the bowel wall (Figure 1). No abscess was identified. In Case 2, no haemorrhage, inflammation or necrosis was observed in the adipose tissue surrounding the perforation site (Figure 2). In Case 3, submucosal haemorrhage was observed, and a thin necrotic tissue layer containing haemorrhage, inflammatory cells, and bacteria was adherent to the bowel wall at the perforation site; however, no suppurative inflammation or necrosis was detected in the surrounding adipose tissue (Figure 3). In Case 4, haemorrhage was observed within the disrupted muscularis propria, and a thin layer of inflammatory cells was present at the base of the submucosa (Figure 4). In Case 5, capillary proliferation with mild lymphocytic infiltration was observed in the submucosa; however, no suppurative inflammation was identified (Figure 5).

## 3. Discussion

The definitive diagnosis of SAIM relies on histological examination [2, 5, 6, 10, 11]. Characteristically, histological sections reveal the absence of the muscularis propria, occasionally accompanied by a thin residual layer. The mucosa and muscularis mucosae remain anatomically intact, and no primary inflammation or necrosis responsible for the perforation is observed in the affected gastrointestinal wall. In our cases, histological examination revealed these characteristic features, although secondary inflammation or haemorrhage associated with the perforation was observed in the area surrounding the perforation site. Therefore, the findings are consistent with those of SAIM. Furthermore, no nerve plexus was identified in the areas where the muscularis propria was absent. In reported cases of SAIM in both newborns and adults, the absence of the enteric plexus in areas of muscularis propria defect has been described in most cases, whereas preservation of the enteric plexus has been reported in others, indicating considerable variability among cases [6, 10]. Accordingly, the presence or absence of the enteric plexus in areas of muscular defect should not be considered a definitive diagnostic feature for SAIM.

Among 86 patients who underwent surgery for intestinal perforation, excluding cases caused by cancer invasion or appendicitis, diverticulitis was the most common cause, followed by SAIM. Likewise, Tsuyuki et al. [11] reported that diverticulitis was the most common cause of intestinal perforation, followed by SAIM; however, they reported a prevalence of 24%, which is considerably higher than the 6% prevalence reported in the present study [11]. Nevertheless, both the present study and the report by Tsuyuki et al. [11] suggested that the prevalence of SAIM as a cause of intestinal perforation may be higher than previously reported. Although diverticulitis is widely recognised as the most common cause of intestinal perforation [12], the relatively high frequency of SAIM remains poorly recognised. Notably, SAIM can only be diagnosed retrospectively. Therefore, in the pathological evaluation of intestinal perforation, it is essential to obtain and examine a sufficient number of tissue sections to avoid overlooking a diagnosis of SAIM. As a routine practice in the evaluation of perforations, we recommend preparing four to six specimens for histopathological examination, including two to four specimens from the perforated area and one from each adjacent non‐perforated wall, all sectioned perpendicularly to the long axis of the perforation. In addition, as noted in a previous case report of a rare colonic lipoma‐associated intussusception [13], both pathologists and clinicians should be aware of the potential risk of intestinal perforation associated with SAIM.

The frequency of SAIM reported by Tsuyuki et al. [11] was 24% (26 of 109 patients with intestinal perforation), which is considerably higher than the 6% observed in our study. In these 26 patients, perforations occurred in the duodenum (*n* = 2), small intestine (*n* = 8) and large bowel (*n* = 16). However, several patients had coexisting conditions that could independently account for the perforation. These included recent surgery for an aortoduodenal fistula (*n* = 1) among patients with duodenal perforation; incarcerated hernia (*n* = 2), recent road traffic trauma (*n* = 2), Crohn’s disease (*n* = 1), recent surgery for colon cancer (*n* = 2) and presumed obstructive colitis secondary to cancer (*n* = 1) among patients with small‐intestinal perforation; and megacolon associated with Parkinson’s disease (*n* = 1), Kayexalate use (*n* = 2), presumed obstructive colitis secondary to cancer (*n* = 2), obstruction due to cancer (*n* = 1) and recent road traffic trauma (*n* = 1) among patients with large‐bowel perforation. Overall, one duodenal case, all eight small‐intestinal cases, and seven of the 16 large‐bowel cases had alternative risk factors that could independently account for the perforation [14–18]. Therefore, assuming a worst‐case scenario in which up to 16 cases were misclassified as SAIM, the estimated frequency would decrease to 9% (10/109). In contrast, among our five cases, only one (Case 5) had a potential alternative cause of perforation (bowel obstruction due to cancer), and the histological findings suggested structural abnormalities of the muscularis propria without involvement of the enteric nerve plexus. These findings indicate that the perforation in our case cannot be solely attributed to bowel wall thinning due to obstruction and support the diagnosis of SAIM. Taken together, the discrepancy in the reported frequency of SAIM between our study and that by Tsuyuki et al. [11] may reflect, at least in part, an overestimation of SAIM in their study cohort.

Nevertheless, the aetiology of SAIM remains unclear. Both congenital factors, including abnormal muscular development during foetal life, and acquired postnatal factors have been suggested [1–5, 7, 8, 10]. Ischaemia has been proposed as a potential contributing factor in both settings. Ischaemic injury can cause necrosis of both the mucosa and muscularis propria; however, because the mucosa regenerates more readily, this may result in a localised absence of the muscular layer [3–5].

Tsuyuki et al. [11] reported that among 26 adult patients with SAIM who underwent surgery for intestinal perforation, the large intestine (from the caecum to the rectum) was affected more frequently than the small intestine (from the duodenum to the ileum), accounting for 16 (62%) and 10 (38%) cases, respectively. The sigmoid colon was the most commonly affected site, accounting for 12 cases (48%). However, as noted previously, a substantial proportion of these cases had alternative aetiologies that could independently account for the perforation. Exclusion of these cases would further skew the distribution towards the large intestine, with the remaining cases predominantly involving the sigmoid colon. Thus, both the original analysis and the re‐evaluated cohort consistently indicate that SAIM in adults occurs more frequently in the large intestine, particularly in the sigmoid colon. Similarly, Nawar and Sawyer reviewed 13 adult patients with SAIM reported in the literature and found that seven cases (54%) involved the colon and six (46%) involved the small intestine [10]. In contrast, neonatal cases of SAIM predominantly involve the small intestine, whereas large intestinal involvement is rare [3, 5, 6]. These differences suggest that the underlying pathogenesis of SAIM differs between neonates and adults.

Clinically, ischaemic colitis predominantly involves the colon, with the sigmoid colon being the most commonly affected site [19]. Although this distribution resembles that observed in adult SAIM, any association between ischaemic colitis and adult SAIM remains speculative, and a direct causal relationship has not been established. Importantly, the present study provides no direct mechanistic evidence linking ischaemia to the development of adult SAIM. Therefore, the role of ischaemia in the pathogenesis of adult SAIM should be interpreted with caution. In contrast, congenital factors are likely to contribute to the pathogenesis of SAIM in neonates.

## 4. Conclusions

Among the 86 adult patients who underwent surgery for intestinal perforation, excluding cases caused by cancer invasion or appendicitis, we identified five cases (6%) of SAIM. Given that the prevalence of SAIM among patients with intestinal perforation may be higher than previously recognised, it is essential to histologically examine adequate tissue samples to avoid missing a diagnosis of SAIM.

## Author Contributions

Concept and design: Naoko Nambu, Nobuyuki Terada; Acquisition, analysis, or interpretation of data: Yuki Kubo, Naoko Nambu, Tsukasa Yoshikawa, Kaito Muroki; Drafting of manuscript: Naoko Nambu, Nobuyuki Terada; Critical review of the manuscript for important intellectual content: Naoko Nambu, Yuki Kubo, Nobuyuki Terada, Shin‐ichi Nakatsuka; Supervision: Nobuyuki Terada, Shin‐ichi Nakatsuka.

## Funding

No funding was received for this manuscript.

## Consent

The requirement for informed consent was waived in accordance with the guidelines of our institution. This study was approved by the Institutional Review Board of Yao Tokushukai General Hospital issued approval (No. 2025‐002).

## Conflicts of Interest

The authors declare no conflicts of interest.

## Data Availability

The data that support the findings of this study are available from the corresponding author upon reasonable request.
